# Reply to Roske and Yeeles: Mismatch correction by a replicative polymerase constrained on DNA by a ring

**DOI:** 10.1073/pnas.2535727123

**Published:** 2026-03-06

**Authors:** Feng Wang, Qing He, Michael E. O’Donnell, Huilin Li

**Affiliations:** ^a^Department of Structural Biology, Van Andel Institute, Grand Rapids, MI 49503; ^b^Laboratory of DNA Replication, The Rockefeller University, New York, NY 10065; ^c^HHMI, The Rockefeller University, New York, NY 10065

**Fig. 1.**
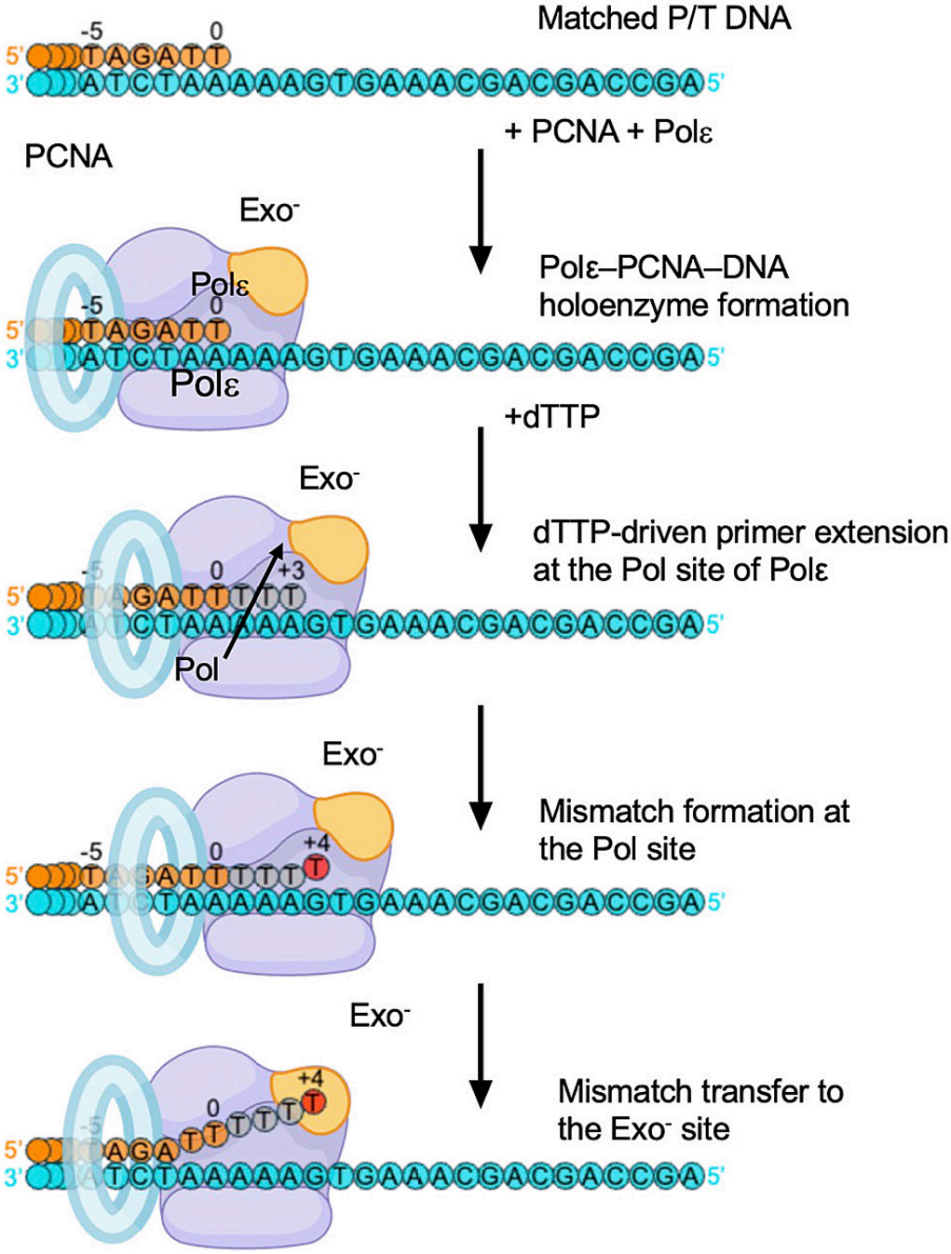
Schematic of in situ mismatch proofreading by Polε–PCNA holoenzyme. The diagram illustrates our strategy (3) for the sequential assembly of Polε–PCNA, DNA extension (3 bp), followed by formation of a 3′ mismatch in situ within the pol site, and finally transfer to the exo site of Polε–PCNA. This is accompanied by 6 bp DNA unwinding required for the switch of the 3′ end from the pol to the exo site while Polε is bound to PCNA.

**Fig. 2.**
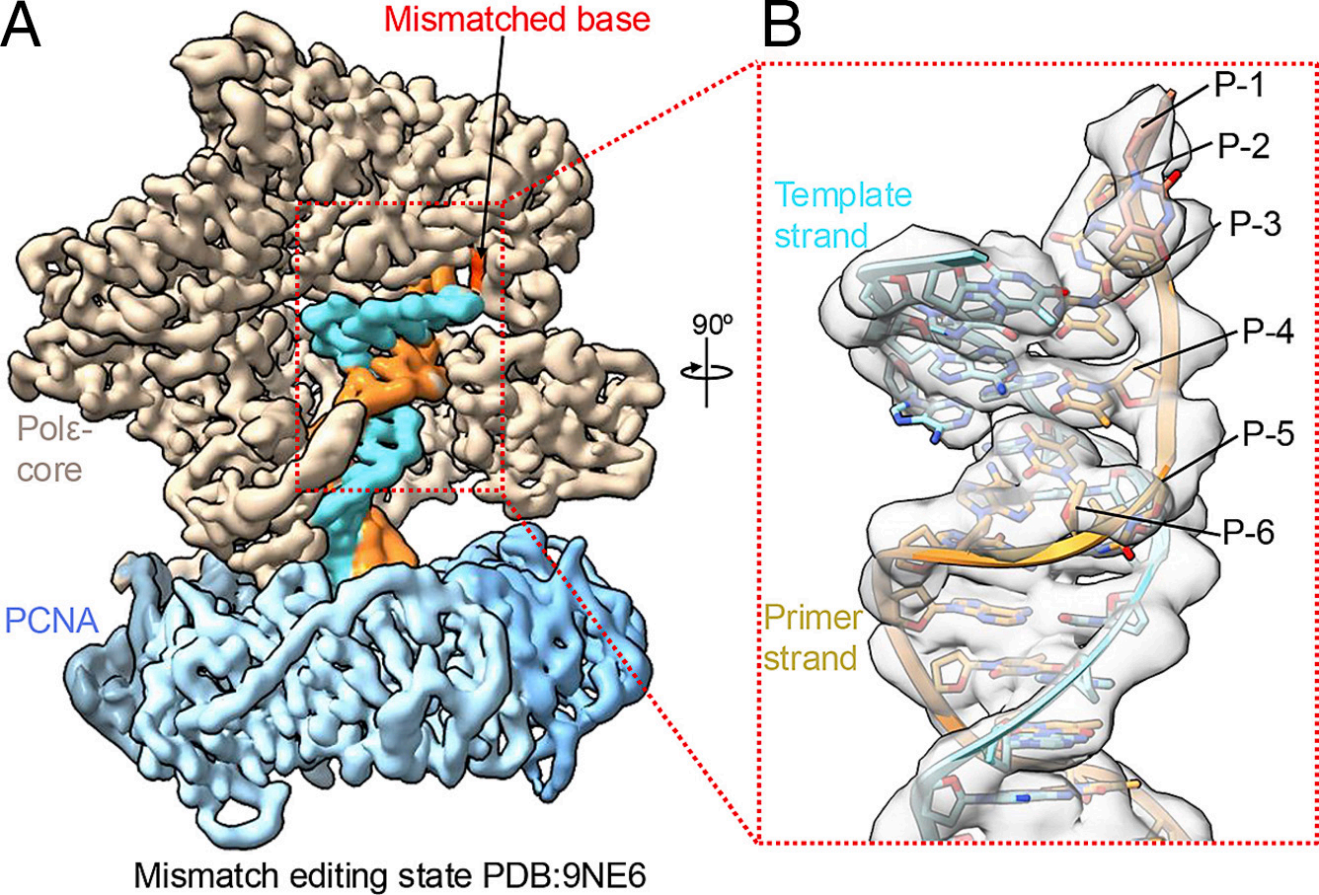
Polε–PCNA unwinds 6 bp of DNA in the mismatch editing state. (*A*) Cryo-EM map of the Polε–PCNA complex in the mismatch-editing state (PDB: 9NE6). The Polε–core, PCNA, and in situ–generated mismatched DNA are shown in different colors. The mismatched base at the primer terminus is highlighted in red. (*B*) Close-up view of the boxed region rotated by 90°, showing the bound DNA density with the template (cyan) and primer (gold) strands. The unwound primer bases (P−1 to P−6) are labeled. Image credit: Reprinted from ref. 3.

Roske and Yeeles comment on our study of 3′ mismatch correction by Polε held on DNA by proliferating nuclear antigen (PCNA) (Fig. 1, figure 2 in ref. 1). Our goal was to observe the constraints that PCNA places on Polε during mismatch correction. Mismatch excision studies generally add a polymerase to a preformed mismatch; the typical result is 3 bp of unwinding for 3′ DNA to enter the exo site. Indeed, addition of Polε to a preformed mismatch (with PCNA added later) yields the 3 bp result, but PCNA is not fully engaged with Polε (*SI Appendix*, figure S5B in ref. 2). These reaction schemes do not require 3′ DNA to first enter the pol site. Therefore, we developed an in situ method that starts with a flush 3′ DNA in the pol site of Polε fully engaged with PCNA; 0.5 mM deoxythymidine-5’-triphosphate (dTTP) is then added to facilitate incorporation of three dTTP’s and a 3′ dT–dC mismatch (+4 dT total), followed by switching of the mismatched 3′ dT to the exo site (Fig. 1) (3). This scheme results in 6 bp unwinding of the 3′ terminus to enter the exo site of a fully engaged Polε-PCNA due to constraints on DNA movements by PCNA (Fig. 2 *A* and *B*). In their recapitulation of our work, Roske and Yeeles show (Fig. 2*B*, lanes 5 to 7 in ref. 1) that the single 3′ mismatch (+4 band) is the major species at 3 min, validating our use of 3-min incubation before grid preparation. A minor +9 band that would require 3 mismatches (figure 2 B and C in ref. 1) is rapidly formed in 10 s, even before the single +4 mismatch. We suggest the rapid formation may be due to undetectable dNTP contaminants largely consumed within 10 s. Some readthrough is eventually expected for exo-deficient Pols (4, 5). We did not use the alternative substrates shown in figure 2D of ref. 1. Importantly, it cannot be known which proteins produce different gel bands, and thus we focused on cryo-electron microscopy (cryo-EM) particles that contained both PCNA and Polε. All three mismatch-proofreading intermediates show density supporting incorporation of the +4 nucleotide, followed by 6 bp unwinding with PCNA fully engaged (Fig. 2).

The other difference between the studies (figure 1 in ref. 1) centers on a Polε–PCNA blunt-end structure, which we do not observe under our conditions. There are numerous differences in the conditions used in the two studies (e.g., T–G vs. T–C mismatch, order of protein addition, buffers, salts, ddATP) (2, 3). While blunt-end binding is not a physiological pathway to proofreading, we agree with their assertion that biotinylation of 5′ ends should specify polymerase binding to a 3′ end.

Reactions in cells are highly dynamic, and proofreading may follow multiple paths. Our study addresses only constraints imposed by PCNA on the exo process. We do not exclude that Polε may associate with a mismatch before PCNA, as in ref. 2. In conclusion, our Polε–PCNA was preassembled on DNA, ensuring mismatch formation in the pol site followed by transition to the exo site. The structural and biochemical data support our original conclusions.
